# Rutin: Family Farming Products’ Extraction Sources, Industrial Applications and Current Trends in Biological Activity Protection

**DOI:** 10.3390/molecules28155864

**Published:** 2023-08-03

**Authors:** Elizabeth Tobar-Delgado, Diego Mejía-España, Oswaldo Osorio-Mora, Liliana Serna-Cock

**Affiliations:** 1 Facultad de Ingeniería y Administración, Universidad Nacional de Colombia, Carrera. 32 Chapinero, Palmira 763533, Colombia; 2 Grupo de Investigación GAIDA, Departamento de Procesos Industriales, Facultad de Ingeniería Agroindustrial, Pasto 522020, Colombia

**Keywords:** antioxidants, extraction, green methodologies, biological activity, nanoencapsulation, plant extracts, phenolic compounds, colloids

## Abstract

In vitro and in vivo studies have demonstrated the bioactivity of rutin, a dietary flavonol naturally found in several plant species. Despite widespread knowledge of its numerous health benefits, such as anti-inflammatory, antidiabetic, hepatoprotective and cardiovascular effects, industrial use of rutin is still limited due to its low solubility in aqueous media, the characteristic bitter and astringent taste of phenolic compounds and its susceptibility to degradation during processing. To expand its applications and preserve its biological activity, novel encapsulation systems have been developed. This review presents updated research on the extraction sources and methodologies of rutin from fruit and vegetable products commonly found in a regular diet and grown using family farming approaches. Additionally, this review covers quantitative analysis techniques, encapsulation methods utilizing nanoparticles, colloidal and heterodisperse systems, as well as industrial applications of rutin.

## 1. Introduction

Traditionally, family farming has presented an important role for society for different reasons: family farming is a relevant provider of food for basic consumption for the population; this employs a significant number of families for productive activities and is the custodian of traditional practices and cultural heritage that have been maintained over time [1].

Family farming accounts for more than 90% of farms worldwide and produces 80% of the food in the world; therefore, in 2017, the United Nations declared the decade of family farming (2019–2028). Through this policy instrument, FAO and the International Fund for Agricultural Development (IFAD) hope to create appropriate environments for family farming in order to secure its position and maximize its contributions to food security and the sustainability of agriculture in the world.

Fruit and vegetable products represent the main food group grown under family farming approaches. These have been traditionally cultivated for culinary purposes whose leaves, stems, fruits, roots and other parts of the plant are a relevant source of dietary micronutrients [2]. However, the global consumption of fruits and vegetables is a challenge regarding food security since the minimum average daily consumption of fruits per person has not yet been reached. Among the reasons why the minimum fruit consumption has not been reached is the lack of knowledge about the nutritional and functional importance of these foods.

Fruit and vegetable production is practiced in many countries as a family farming model, mainly in developing countries; fruit and vegetable production contributes significantly to the economy and population health [3]. According to a report from the Food and Agriculture Organization (FAO, 2020) [4], it was estimated that around 800 million people practice family farming, prioritizing the typical fruit and vegetable crops of each region. In the case of countries in the Andean region, legume, root and tuber crops were relevant; all of these have exceptional nutritional characteristics and biological value compounds. However, current research is focused on the study of new sources of biocomposites, neglecting research related to accessible food and easy management of crops.

Rutin, (3′,4′,5,7-tetrahydroxy-flavone-3-rutinoside), also known as vitamin P, rutoside, quercetin-3-O-rutinoside and sophorin, is a flavonol glycoside that was first detected in *Ruta graveolens*, commonly known as rue, and has been found naturally in some commonly consumed plant species [5,6]. Rutin has wide relevance to the scientific field due to its pharmacological potential. A lot of reviews have reported about its anti-inflammatory, antidiabetic, cardiovascular, hepatoprotective, anticancer and neuroprotective activity [7,8,9,10,11].

Other reviews have compiled information about rutin extraction sources from medicinal plants and exotic fruits, whose cultivation method depends on the conditions specific to the geography of each region. It has also been established in the literature that correct comparisons between extraction methods are difficult due to the variance in plant origin and extraction conditions [12,13]. However, research related to foods for daily consumption, that is, foods that can be easily grown in family settings and complement food security approaches, has often been overlooked. The routine can be obtained through fruits and vegetables for daily consumption that are grown in family farming.

Rutin extraction from horticultural foods is relevant for fundamental research and, subsequently, for future applied research. However, the industrial application of rutin has remained challenging due to the physicochemical characteristics of rutin, such as its low solubility due to its configuration of phenolic rings; therefore, it cannot be absorbed using a simple diffusion process. Furthermore, rutin has little miscibility with lipids, which limits solubility in the cell membrane [7]. One way to reduce this limitation and, consequently, increase the action of the compound is based on encapsulation methods, where structures loaded with the active compound are designed and the formulation characteristics depend strictly on the encapsulated active.

Different research has been published on the encapsulation of rutin with polymers and lipids to improve stability and solubility. Encapsulation techniques include spray drying, coacervation, liposome entrapment, nanoemulsions, complexation, co-crystallization, nanoencapsulation and aqueous lyophilization, among others [14,15,16,17,18,19,20]. These investigations, mostly in vitro industrial applications of both a pure rutin compound and an encapsulated extract, are limited, with priority given to pharmacological formulations, while food applications have been neglected. Therefore, this review focuses on informing readers about the sources of rutin extraction, emphasizing foods grown under agricultural family and community practice approaches. Current trends in extraction techniques and protection of the biological activity of rutin through the study of colloidal and heterodisperse systems are presented. In addition, we include a chapter related to research on rutin’s application in different industrial environments.

## 2. Materials and Methods

Web of Science (WOS) databases were used to collect the literature in this review. Databases such as: Web of Science Core Collection, Data Citation Index, SciELO Citation Index and Science Direct were searched. Filters were applied to select studies published in the last ten years. For the information search, the following combinations of terms were preferred: “flavonol name” and keywords such as: “horticultural sources”, “extraction”, “encapsulation”, “heterodisperse systems”, “food formulations” and “pharmaceutical formulations”.

Databases were also searched using keywords such as “flavonol name”, “release” and “in vitro assay”, “in vivo assay” to collect data on the current status of industrial applicability of rutin flavonol.

## 3. Structure and Physicochemical Properties of Rutin Flavonol

**Figure 1:**
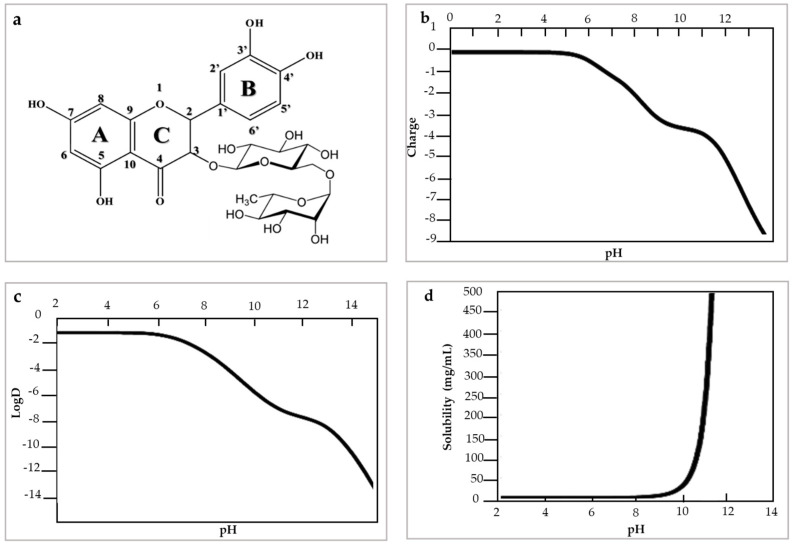
Chemical structure and physicochemical properties of rutin flavonol, modified from [21] (**a**) Chemical structure, (**b**) rutin charge, (**c**) hydrophobicity (logD), (**d**) rutin solubility at different values of pH.

Physicochemically, rutin has a molecular weight of 610.518 g/mol, pKa: 4.3 and log *p* value: −1.97 measured in acetonitrile at 50 °C. Rutin has poor water solubility in acidic and neutral environments but has greater solubility in an alkaline environment. This condition is due to the change in its electric charge and hydrophobicity (log D) with the change in pH [21] (Figure 1). Rutin has a strong negative charge and is highly hydrophilic under alkaline conditions, and is uncharged and slightly hydrophilic under acidic conditions [9]. The low solubility of rutin has limited its industrial applications. This condition is related to the ring structures, which are too large to be absorbed via a simple diffusion process [22].

The route for the synthesis of rutin is via the phenylpropanoid route [6]. Phenylalanine is transformed into cinnamic acid for the action of the ammoniacal phenylalanine enzyme, then cinnamic acid is catalyzed to form coumaric acid and then 4-coumaryl CoA by 4-coumarate: CoA ligase. The chalcone is made up of 4-coumaryl CoA. Subsequently, calchona isomerase catalysis leads to naringenin and the flavonol is formed under flavanone 3-hydroxylase catalysis. Flavonol is catalyzed to dihydroquercetin, quercetin formation comes from flavonol synthase catalyzing dihydroquercetin and finally rutin is formed by quercetin under the action of glycosyltransferase and two-step glycosylation [23,24].

## 4. Sources of Rutin Obtained in Family Farming Products

Fruits and vegetables are a fundamental component of the regular diet and are among the most commonly consumed products worldwide. They are generally affordable and represent traditional agricultural practices within communities and families [25]. Although global initiatives such as the declaration of 2013 as the international year of Quinoa have highlighted the health benefits associated with the consumption of certain crops, many other fruit and vegetable crops have yet to receive comparable attention in terms of global consciousness [26].

Many consumers are unaware of the presence of bioactive compounds in fruits and vegetables. While nutritional components such as proteins and carbohydrates often receive the most attention, the added value of phytochemicals and other bioactive compounds in promoting health is frequently overlooked. It is crucial to disseminate information about the beneficial properties of these compounds to raise awareness and promote the consumption of fruits and vegetables as staples of a balanced diet. By means of identifying rich sources of phytonutrients and exploring appropriate extraction methods for these compounds, the production, marketing and consumption of horticultural products can be improved to benefit all sectors of the production chain.

Antioxidants such as rutin flavonol are found in fruit and vegetable products of plant species including: *Polygonaceae*, *Solanaceae*, *Capparaceae*, *Amaranthaceae*, *Asteraceae*, *Celastraceae*, *Asparagaceae*, *Chenopodiaceae*, *Lamiaceae* and *Rosaceae* [27,28,29,30]. In addition, the search for sustainable sources of rutin extraction allowed the study of raw materials generated from agro-industrial waste. Rutin has been found in stems and calyx of fruits; these have been found after the harvest of varieties such as Physalis peruviana, some of the genus Fagopyrum and in banana leaves [31,32,33].

Leafy vegetables like lettuce are regularly consumed in salads and are a source of rutin. A rutin content of 750.82 µg/g has been reported in lettuce leaves, which varied in phenolic compounds depending on the season of cultivation, with winter-grown lettuce showing the highest rutin content [34]. Different parts of the lettuce plant have been studied to quantify rutin. For instance, it was found that the roots of lettuce had a higher rutin content (172.09 μg/g) than the leaves in the vegetative stage [35]. Meanwhile, a rutin content of 128 µg/g was reported during the harvest stage [36].

The content of rutin in species of the Brassicaceae family, such as broccoli, has been investigated to promote their consumption. In this case, rutin was quantified in broccoli stored in bulk and a concentration of 102.14 µg/g was found [37]. The authors linked the presence of antioxidant compounds in broccoli with health benefits, such as the prevention of degenerative diseases. Within the same Brassicaceae family, cauliflower sprouts were studied to quantify rutin, and the authors reported concentrations of 300 µg/g, a concentration close to the daily recommended intake of this type of compound [38].

Fruits and vegetables that belong to the Cucurbitaceae family, namely watermelon (*Citrullus lanatus* L.), pumpkin (*Cucurbita maxima* L.), cucumber (*Cucumis sativus* L.) and melon (*Cucumis melo* L.), are globally significant crops often utilized as salad ingredients, juice bases, desserts and in other culinary preparations. Additionally, melon has been traditionally used to treat liver inflammation, coughs and kidney disorders, such as urinary tract ulcers; it has also been indicated as a source of rutin [39]. Table 1 displays the quantified rutin concentrations found in this type of produce.

The Chenopodiaceae family includes several significant tubers among fruit and vegetable crops, with beets being an example. In the culinary industry, beets are recognized for their striking purple–red hue and are incorporated into salads, entrees such as chips and purees and others. Additionally, beets are a rich source of nutrients that include complex B and C vitamins, minerals, fiber, protein and bioactive phenolic compounds such as betalains. Flavonoids such as rutin, kaempferol, rhamnetin and astragalin are among the most significant found in beets [40]. Pharmacologically, beetroot has been found to exhibit antioxidant, antimicrobial, anticancer, hypocholesterolemic and anti-inflammatory properties [41].

On the one hand, aromatic plants are often used in the culinary industry as seasonings and condiments to enhance the sensory properties of food. Additionally, these plants have been cultivated by humans since ancient times and are widely used in the pharmaceutical and agricultural industries as a source for treating a range of disorders. Aromatic plants contain active compounds that make them useful for treating physical and mental ailments, such as having anti-inflammatory, anti-infectious and sedative properties, among many others. They are effective against influenza, gastrointestinal disorders, anxiety, seizures, rheumatic pain, muscle spasms, ulcerations and hemorrhoids, and function as antiseptics, disinfectants, bactericides and fungicides [42]. Aromatic plants are known for their ability to thrive under a wide range of growth conditions and are relatively easy to cultivate. Furthermore, they play a natural role in pest protection for nearby crops, making them an ideal choice under family farming approaches. Therefore, orchards that feature a diverse array of aromatic plants are commonly promoted [43].

Several studies have reported the extraction and quantification of rutin in various aromatic species. For instance, researchers extracted rutin from calendula using an ultrasound-assisted technique and reported a yield percentage of 2.28% (*p*/*p*) [44]. In a separate study, a rutin content of 8.9% was demonstrated in oregano using hydroethanolic extraction [45]. Furthermore, basil was found to contain a rutin concentration of 15 mg/100 g, while coriander exhibited a significantly higher concentration of 115 mg/100 g [46].

Consequently, efficient extraction methods of rutin involve modeling and optimizing operational variables, in addition to the use of safe enzymes and solvents that allow for selective extraction. Due to the rutin flavonoid’s skeletal structure and numerous hydroxyl groups, protic compounds like ethanol, glycerol or 1,3 butanediol are often used to extract rutin. The extraction process is handled at temperatures ranging from 30 to 70 °C as reported [47].

Alternative technologies such as high pressure, (supercritical fluids, pressurized liquid), ultrasound, microwave and emerging methodologies such as NADES are reported in current research on rutin extraction.

### 4.1. Microwave-Assisted Technology in Rutin Extraction

The microwave-assisted extraction technique (MAE) is emerging as a promising alternative to conventional extraction methods. In 1986, Gedye and Giguere introduced microwave energy in organic synthesis as a novel technique. Ganzler applied (MAE) in the extraction of biological matrices for the preparation of analytical samples [48].

Microwaves are a form of non-ionizing electromagnetic radiation that oscillates perpendicularly at frequencies ranging between 300 MHz and 300 GHz [49]. Owing to their non-ionizing nature, microwaves interact with plant matrices primarily through the transfer of heat caused by ionic conduction and dipole rotation. This selective process is dependent on the dielectric constant of the material being heated [35].

The microwave-assisted method has been redesigned for different methods to adapt to other types of extraction. These changes have allowed for adaptations to vacuum extraction, nitrogen-protected extraction, microwave-assisted ultrasonic extraction and solvent-free extraction. As a result, two types of equipment systems can be differentiated in the market: open and closed microwave systems [50].

**Figure 2:**
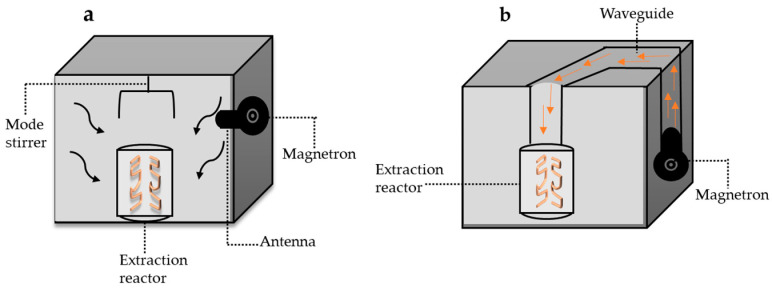
Microwave-assisted extraction equipment, modified from [53] (**a**) closed microwave system, (**b**) open microwave system.

Closed microwave equipment utilizes the parallel or perpendicular scattering of microwave radiation around the entire cavity as shown in Figure 2a; this closed system is used to adapt the technique to high pressure. On the other hand, the open system allows for microwave radiation to occur in a specific part of the cavity, as shown in Figure 2b; in turn, this system is generally used to work under atmospheric operating pressure [51].

Rutin extraction using the microwave-assisted technique has been carried out using solvents such as ethanol and water. The extraction of compounds of bioactive compounds from fig leaves was studied. The solubility studies demonstrated that non-ionic solvents had higher solubility in relation to the metabolites to be extracted; thus, the presence of several hydroxyl groups in the surfactant could trigger strong hydrogen bonding with target compounds, making them easier to be captured [52]. The optimized conditions of the research included a liquid–solid ratio of 19.95 mL/g, extraction temperature of 39.53 °C and extraction time of 10.25 min, power of 400 w and polyethylene glycol 8000 solvent; the rutin of fig leaves resulted in an extraction yield of 4.77 mg/g. Table 1 shows research related to rutin extraction from horticultural products using the microwave-assisted technique.

### 4.2. Ultrasound-Assisted Technology in Rutin Extraction

**Figure 3:**
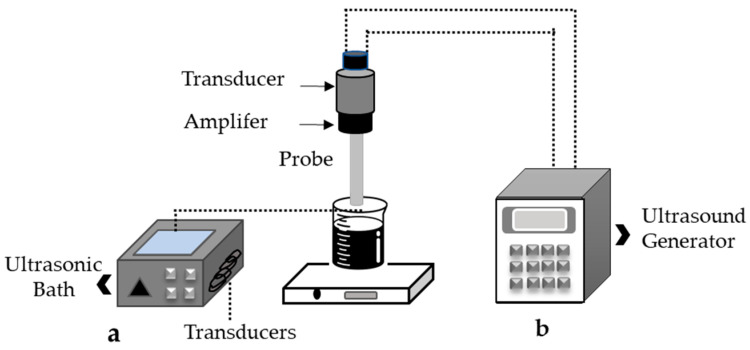
Ultrasound equipment, modified from [56] (**a**) Ultrasonic bath (**b**) sonication probe.

Ultrasound is a technology used to assist with the extraction of biological compounds and is considered an efficient and economical alternative to traditional methods [54]. The sonication process involves the transfer of ultrasonic waves (>16 kHz) in a liquid medium, which leads to cavitation phenomena. This creates, grows and collapses gas bubbles due to pressure differences and expansion/compression cycles that occur during the process, and facilitates the extraction of compounds (Figure 3).

The use of low frequencies less than 100 kHz and high-power intensity (10–1000 W/cm^2^) during sonication produces localized high temperatures (<4727 °C) and pressures below 101 MPa due to the collapse of bubbles, resulting in high shear rates in solids and surfaces [55].

Research has been conducted to compare the ultrasound-assisted technique with other techniques in rutin extraction. In the study conducted by [57], conventional extraction techniques were compared with ultrasound-assisted extraction. The results showed a higher percentage of rutin extraction with ultrasound treatment for 6 min at 55 °C and an amplitude wave of 40%; additionally, significant differences were observed in the extraction yield and better antioxidant capacity with the sonication treatment.

Ultrasonic treatment application promotes the disintegration of cells by shear force, thus allowing the release of the compounds of interest to be subsequently extracted by the solvent. According to bibliographic reports on the extraction of the compound rutin, sonication times between 5 and 30 min have been used according to the type of equipment, frequency and power used [58,59,60].

Optimization tools are commonly used in ultrasound-assisted extraction processes to improve efficiency. Parameters such as wave amplitude, power, frequency, sonication time and temperature, among others, are frequently studied. The optimization tools must enable the simultaneous analysis of variable effects, optimization of the experimental conditions and a reduction in the number of experiments required. However, research on the extraction of the rutin flavonol using ultrasound-assisted techniques remains mostly at a laboratory scale, prioritizing kinetic mathematical modeling of the degradation compound. Table 1 shows research related to rutin extraction from horticultural products using the ultrasound-assisted technique.

### 4.3. Supercritical Fluid Technology in Rutin Extraction

Any type of substance, whether gas or liquid, that is above its critical temperature and pressure is classified as a supercritical fluid (SF). At this point, there are no distinct gas or liquid phases, and a homogeneous fluid emerges [61].

Physiochemically, the density and diffusivity of a supercritical fluid (SF) changes with slight modifications in the operating parameters, such as pressure and temperature, resulting in SF exhibiting properties between those of a solid and gas with increased solvent capacity. This selectivity allows SF to diffuse through a complex matrix, extracting specific compounds of interest [62].

Although different solvents, including water, ethane, propane, butane and ethanol, can be used for extraction under supercritical conditions, CO_2_ is the most commonly used solvent due to its benefits. This solvent has a low critical temperature and pressure values (32 °C and 7.4 MPa, respectively), making it suitable for various extractions. Additionally, it is non-toxic and highly pure; CO_2_ can be easily removed from the extracted product since it is a gas at room temperature and atmospheric pressure. The use of CO_2_ as a solvent has been estimated to be involved in about 90% of various extraction processes [63].

In supercritical rutin extraction, an ethanol/water mixture is commonly used as a solvent. the addition of water increases the density of the mixture, which causes solid particles to swell, leading to an improved internal diffusion process that enhances the solubility of the compounds to be extracted [64].

The provided search results indicate that while most rutin extraction studies use combinations of organic solvents, in a research study, supercritical water was used for semi-continuous extraction of rutin from buckwheat and achieved a yield of 91.0% at 120 °C and 3 mL/min. Additionally, for the extraction of rutin from asparagus, the ratio of ethanol/water cosolvents to supercritical CO_2_ was studied; the researchers obtained a comparable extraction yield of 42.6% at 15 MPa and 65 °C. The authors attribute the extraction yields to the affinity of the OH substituents of the rutin with the polar solvents (ethanol/water mixture) [65].

Supercritical fluid extraction techniques require optimization of the operating conditions to achieve the conditions for mass transfer, surface tension and dielectric constant [66]. Therefore, modifying any operational variable, such as pressure, which has significant impact on the process, may alter the selectivity of carbon dioxide in the supercritical state, ultimately affecting the chemical profile of the resulting extracts [67].

The supercritical fluid extraction process involves CO_2_ in the storage cylinder which is pumped through a high-pressure pump. The liquid CO_2_ is then compressed to the appropriate process temperature using a medium from the high-pressure pump, based on the required pressure. To improve supercritical CO_2_ selectivity, polar solvents or a mixture of polar solvents may be added [27].

**Figure 4:**
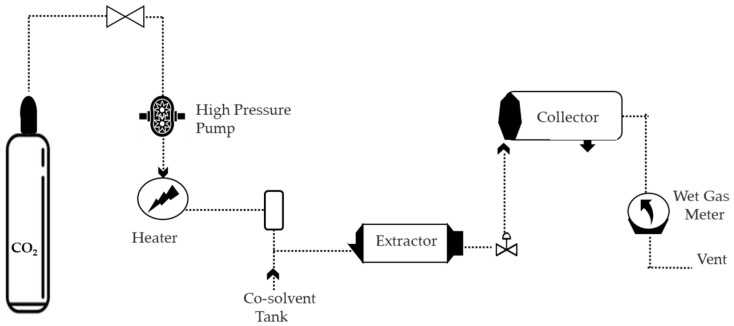
Supercritical fluid technique, schematic of the extraction process, modified from [64].

Afterwards, the supercritical CO_2_ flows into an extraction vessel containing the plant material. The CO_2_ enters the depressurization valve, causing the compounds of interest to precipitate and allowing them to be collected in a separator [64], as shown in Figure 4.

### 4.4. Alternative Solvents for Rutin Extraction

Natural deep eutectic solvents (NADES) are obtained from primary metabolites such as amino acids, organic acids, sugar or choline derivatives, and can exist in solid or liquid form. However, NADES in particularly designed combinations or molar ratios have an optimum melting point and become liquid at room temperature, decreasing their viscosity and promoting intermolecular interactions like hydrogen bonding or ionic bonding [68].

**Figure 5:**
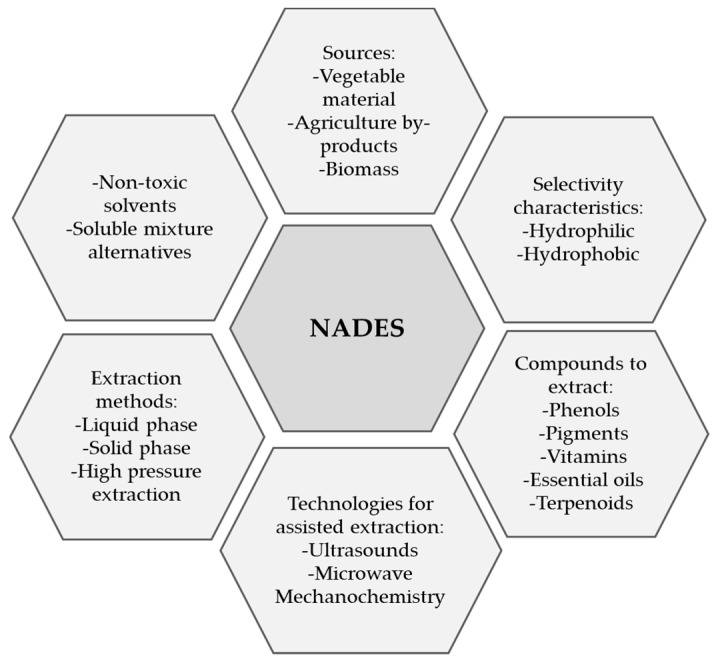
Main advantages of natural deep eutectic solvents (NADES), modified from [72].

Hydrogen-bond acceptor compounds in NADES are typically amines or amino acids, whereas hydrogen-bond promotors can be organic acids or carbohydrates such as glucose, fructose and maltose [69]. Extraction with NADES provides several advantages, as shown in Figure 5, including the ability to work at low pressures and the low flammability of the solvents. NADES have a low melting point and even remain in a liquid state below −20 °C. Their mixtures are easy to purify and prepare. In addition, NADES are highly effective at solubilizing low-polarity compounds [70,71].

The rutin flavonol was extracted from *Sophora japonica* using a plant using NADES. The researchers utilized a NADES mixture of choline chloride and glycerol in a 1:1 molar ratio and obtained an extraction efficiency of 291.57 mg g^−1^ without presenting significant differences with respect to the extraction results with the methanol solvent.

In the same research line, NADES components were used in combination with ultrasound, and it was demonstrated that the incorporation of choline chloride and glycerol- significantly increased the solubility of rutin. The researchers reported a maximum of 9.5 mg/g of rutin in the extract, with an extraction efficiency of 95%. In addition, minimal toxicity and favorable biodegradation for the extraction solvents were reported [73].

For the extraction of rutin from *Sophora japonica* flowers, NADES were used. The mixture of choline chloride and triethylene glycol containing 20% water favored the solubility of the material, achieving an extraction efficiency of 194.17 ± 2.31 mg/g^−1^. As a complement to this study, a biocompatibility evaluation against various bacteria including *E. coli*, *S. enteritidis*, *S. aureus* and *L. monocytogenes* was performed. The results showed that the NADES used presented greater biodegradability, reaching up to 69% [74].

Table 1 presents supplementary research pertinent to various sources of extraction, extraction methods and the rutin content in the extracts.

**Table 1:** Table summarizing family farming products as sources of rutin extraction, methodologies and extraction results.

Rutin Extraction Source	Extraction Method	Process Variables	Rutin Concentration	Ref.
Thyme (*Thymus serpyllum* L.)	Lixiviation	Methanol solvent, 5:50 (*w*/*v*), 24 h, 30 °C	875 μg/g	[75]
Lettuce (*Lactuca sativa*)	Maceration	Methanol solvent, 1:5 (*w*/*v*), 3 h	0.186 a mg/g	[76]
Lettuce (*Lactuca sativa*)	Maceration	Methanol solvent, 0.5:10 (*w*/*v*)	50 mg/100 g	[77]
Cauliflower *(Brassica oleracea var. botrytis*)	Maceration, filtration	Methanol solvent, 25:250 (*w*/*v*)	551 μg/g	[78]
Cucumber (*Cucumis sativus* L.)	Soxhlet	Ethanol solvent 70%, 30, 3 h	647.08 µg/g	[79]
Thyme (*Thymus vulgaris*)	Soxhlet	Ethanol solvent 70%, 4 h, 50 °C	10.05 mg/mL	[80]
Strawberry leaves	Microwave-assisted extraction	Ethanol solvent (51.1%), 6:61 (*w*/*v*) microwave power of 300 W	8.08 mg/g	[81]
Broccoli (*Brassica oleracea* L.)	Maceration	Methanol solvent (80%), 2 h, pH:2	55.35 μg/mg	[82]
Eggplant	Lixiviation	Solvents: n-hexane, dichloromethane, ethanol, 95% (*v*/*v*) 1:10 (*w*/*v*), 30 °C	32.4 mg/g	[23]
Tarragon (*Artemisia dracunculus*)	Soxhlet	Methanol solvent 200:10 (*w*/*v*), 48 h	610 mg/100 g	[46]
Sophora *(Sophora japonica)*	NADES	Choline chloride and glycerol (1:1)	284.81 mg/g	[83]
Cassava leaves (*Manihot esculenta Crantz)*	Ultrasonic-assisted extraction	Ethanol (60%), 50 °C, 2.5:50 (*w*/*v*), frequency 40 kHz 90 min	24.49 g/kg	[60]
Rosemary	Ultrasonic-assisted extraction	Methanol solvent 80%, formic acid (1%), 2:20 (*w*/*v*), 50 °C, 10 min	378 μg/mg	[84]
Cassava (*Manihot esculenta Crantz*)	Ultrasonic-assisted extraction	Ethanol solvent (50%), 1:40 (*w*/*v*), 50 °C, 4 h, power: 80 W	622 mg/100 g	[85]
Golden berry leaves (*Physalis peruviana* L.)	Lixiviation	Ethanol solvent (50%), 1:10 (*w*/*v*), 60 °C, 5 h	4996.37 μg/g	[86]
Golden berry (*Physalis angulata*)	Ultrasonic-assisted extraction	Solvents: 57% of water, 35% of ethanol and 8%, 0.6:15 (*w*/*v*), 10 min, power: 90 W, 30 °C	88.2 μg/g	[29]
Chili pepper (*Capsicum annuum* L.)	Maceration	Ethanol solvent 70% *v*/*v* acidified by HCl (pH = 3), 2 h	2.76 μg/mg	[87]
Broccoli (*Brassica oleracea* L.)	Decoction	Methanol solvent (60%), 500:20 (*w*/*v*), boiled for 5 min	0.09 mg/kg	[88]
Black carrot (*Daucus carota* L.)	Maceration	Solvents: methanol, water, acetic acid (70/29.5/0.5, *v*/*v*/*v*), 35:150 (*w*/*v*), 2 h	0.075 mg/100 g	[89]
Sideritis condensate	Maceration	Ethanol solvent 100 °C, 30 min	879 μg/g	[90]
Cabbage (*Brassica oleracea sabauda*)	Soxhlet	Ethanol solvent (50%), 25:50 (*w*/*v*), 24 h	129.97 μg/mg	[91]
Tartary buckwheat (*Fagopyrum tataricum*)	NADES	Choline chloride and glycerol 40:1 (*w*/*v*), 40 °C, 1 h	9.5 mg/g	[73]
Amaranth *Amaranthus paniculatus*	High-pressure extraction	Solvents: 70:30 (*v*/*v*) water/ethanol, 188 °C, 20 min, 10 MPa	14.30 g/kg	[92]
Radish (*Raphanus sativus* L.)	Maceration	Methanol solvent (80%), 5:20 (*w*/*v*), 30 min	9.15 mg/100 g	[93]
Cucumber (*Cucumis anguria* L.)	Ultrasound-assisted extraction	Methanol solvent, (80%), acetic acid (1%), 0.1:10 (*w*/*v*), sonication time: 30 min, 1200 rpm	205.10 µg/g	[94]
*Solanum tuberosum*	Ultrasound-assisted extraction	Methanol solvent (40%), 3:25 (*w*/*v*), sonication time: 15 min	10.76 mg/kg	[95]
Celery *(Apium graveolens)*	Soxhlet	Methanol solvent, 10:100 (*w*/*v*), 30 min, 40 °C	417 mg/100 g	[96]
Lettuce (*Lactuca sativa*)	Soxhlet	Methanol solvent, 1:10 (*w*/*v*), 4 h	78.43 μg/mL	[97]
Broccoli (*Brassica oleracea* L.)	Ultrasound-assisted extraction	Methanol solvent, 5:10 (*w*/*v*), sonication time: 15 min, 2700 rpm	0.44 a mg/g	[98]

## 5. Analytical Techniques for Qualitative and Quantitative Analysis of Rutin

Analytical techniques can be used to identify and quantify rutin compounds. The sensitivity of detection limits and the ability to detect similar metabolites or degradation products depend on the specific technique used. Recently, studied techniques include high-performance liquid chromatography (HPLC), mass spectrometry-coupled liquid chromatography, gas chromatography, electrochemistry, fluorescence methods, UV-VIS spectrometry and Fourier transform spectroscopy (FT-IR) [99].

### 5.1. HPLC and Liquid Chromatography Coupled to Mass Spectrometry

High-performance liquid chromatography (HPLC) is a reliable technique for rutin’s qualitative and quantitative determination due to its high repeatability and reproducibility. Some research has studied mobile phases containing 0.5% acetic acid and acetonitrile, resulting in faster analysis, better resolution and high linearity (useful for quantification), and specificity. This method allows one to detect rutin levels greater than 0.38 parts per million with a measurement time of approximately 15 min and a required sample volume of 5 µL. The detection is performed using a UV light reader at 356 nm while keeping the sample temperature at 26 °C [100].

Liquid chromatography coupled to mass spectrometry combines the compound-separation capabilities of liquid chromatography with the high sensitivity of mass spectrometry to detect and quantify compounds in complex mixtures. This technique is highly sensitive (<2 nM/L) and reproducible, making it a valuable tool for a wide range of applications. In rutin detection, 0.1% formic acid and acetonitrile have been used as the mobile phase, enabling efficient compound separation and detection [101].

### 5.2. Electrochemical Methods

Electrochemical methods offer a rapid, precise and cost-effective means of detecting compounds of interest, without requiring any alteration of the sample. These methods rely on the electrochemical interaction between two compounds and generally involve a device with two electrodes that interact with the analyte, inducing either oxidation or reduction of the substance. This process generates a response in terms of voltage, current, resistance or charge, which can be interpreted as a concentration of the substance of interest. Devices for the electrochemical measurement of substances, such as biosensors, have shown great promise in a wide range of applications [102].

Flavonols are electrochemical compounds that are well-suited for detection using electrochemical methods due to the presence of a hydroxyl group that enables their oxidation or reduction in the presence of different electrodes [103].

Platinum nanoparticles and graphene nanocomposites are capable of detecting rutin at concentrations as low as 0.02 µM, while modified electrodes made from carbon nanotubes and gold nanoparticles can detect rutin at concentrations as low as 0.81 nM [104].

Some materials have been used and altered to detect rutin through electrochemical means. These materials include glassy carbon electrodes, glassy carbon electrodes modified with graphene oxide, glassy carbon electrodes modified with amine-functionalized Fe_3_O_4_ nanoparticles [103] and pyridine-modified carbon paste electrodes, which have a detection limit of 3.58 × 10^−7^ M [105].

### 5.3. Fluorescence Methods

Measuring the concentration of rutin can be challenging due to the lack of selectivity in the measurement processes, particularly in the presence of other flavonoids like quercetin. However, researchers have developed analytical techniques utilizing nanoparticles to improve selectivity during the measurement process. These techniques include the use of silicon dispersed in water and bovine serum albumin, which have shown promise in improving the accuracy and specificity of rutin detection.

Silicon nanoparticles naturally exhibit a decrease in fluorescence in the presence of flavonoids. However, the addition of bovine serum albumin enables selectivity in the process, whereby only the rutin particles experience a fluorescence decrease, allowing for its detection and quantification. This is due to the high affinity that bovine serum albumin has with compounds like quercetin, which acts as a chelating agent, reducing interference. This technique permits measurements of rutin concentration ranging from 0.33 to 33.33 mM. Further, to ensure high specificity in the process, the detection limit is 0.04 mM [99].

The spectrofluorometric determination of rutin has been demonstrated using activators such as hemoglobin catalase. This method promotes a reaction between the enzyme, hydrogen peroxide and nicotinamide-adenine dinucleotide (NADH), exciting NADH at a wavelength of 340 nm and measuring fluorescence at 450 nm. The changes in the fluorescence signal of the fluorophore are primarily due to the presence of rutin, which can be quantified and related to the amount of analyte present in the sample. The detection limit of this technique is 7 × 10^−8^ mol/L [106].

### 5.4. UV-VIS Spectrometry and FT-IR Spectroscopy

Vibrational techniques such as FT-IR, combined with electron spectroscopy such as UV-VIS, have been used as a tool for rutin analysis. Vibratory techniques are useful for rutin identification in the presence of impurities, while electron spectroscopy allows for the evaluation of changes in the compound resulting from various physicochemical factors such as changes in temperature, pH and the presence of degrading agents, among others. This technique can identify rutin in the presence of impurities, as well as its degradation compounds, which makes it possible to assess the stability of the compound over time [107].

## 6. Current Trends to Preserve Therapeutic Properties and Improve the Efficiency of Rutin

The presence of hydrophilic hydroxyl groups and a lipophilic benzene ring in the structure of rutin results in its poor solubility in water, which limits its direct application in food, pharmaceutical and cosmetic products. Other reported issues with rutin include a short shelf life, physicochemical and organoleptic deterioration due to environmental stress or the mixture of compounds during processing, uncontrolled release, instability under digestive conditions in the gastrointestinal tract and low bioavailability.

Some researchers have investigated plant matrices with high levels of rutin, as well as pure rutin, to study encapsulation systems that protect its biological activity. Currently, nanoscale technology approaches are at the forefront of these investigations, which the industry has adopted to enhance the solubility of lipophilic compounds, encapsulate food additives, achieve controlled release under gastrointestinal conditions and develop specific drug delivery systems [108].

### 6.1. Rutin Encapsulation in Colloidal and Heterodisperse Systems

While rutin encapsulation techniques have yielded promising results for preserving the biological activity of various compounds, the slightly lipophilic properties of rutin make it suitable for encapsulation using nanoscale techniques based on colloidal and heterodisperse systems incorporating lipids or polymers. Among these techniques are phospholipid complexes, phytosomes, liposomal systems, nanoemulsions and lipid and biopolymeric nanoparticles, which are all examined in this review [109,110,111].

Design structures with nanometer-sized particles associated with colloidal systems inherently represent a non-spontaneous formation process where the change in Gibbs free energy is positive and, therefore, the application of external energy is required to adjust the particle size and prolong the stability of the system [112]. In addition, the reduction in size generates an increase in the surface area that favors the solubility of lipophilic compounds without neglecting force phenomena that originate, such as Brownian movement, which is more significant than gravity and, consequently, would have greater kinetic stability due to a weaker gravitational force [112].

The stability of colloidal systems is defined by the balance of attractive and repulsive forces and steric hindrance. According to the Deryagin–Landau–Verwey–Overbeek (DLVO) theory, the surface charge must maintain a distance from neutrality to keep a colloidal system stable [113]. Calculable parameters like the polydispersity index and Z-potential can be used to estimate stability in nanostructured colloidal systems via electrophoretic and electroacoustic methods.

Finally, Z potential calculation helps estimate surface charge based on the electrophoretic movement of particles [114]. It is essential to select system components carefully to modulate the Z potential values. This involves identifying the type of surfactant to be used, incorporating it into the continuous phase interface and selecting compounds to adsorb on the structural surface, such as biopolymers or chemical compounds of interest to be encapsulated [115].

#### 6.1.1. Liposomal System

Liposomes are commonly defined as spherical vesicles with a particle size ranging from approximately 50 nm to a few microns. Liposomes consist of an aqueous core or center surrounded by one or more lipid bilayers of either natural or synthetic origin [116]. Liposomal systems are formulated by mixing aqueous substances with dipolar compounds such as phospholipids, which have amphipathic characteristics allowing them to form vesicle-shaped structures when hydrated in an aqueous medium [117]. There are different classifications of liposomal systems, but a general classification includes small unilamellar vesicles, large unilamellar vesicles, giant unilamellar vesicles and multilamellar vesicles [118].

The encapsulation of phenolic compounds involves the design of liposomal systems that specifically bind functional flavonoid groups with lipid compounds. To this end, the chemical structure, analyte concentration, composition of lipid molecules (including the use of cholesterol and ionic lipids) and incorporation of synthetic or natural biopolymers are commonly studied factors [119].

According to the research, due to the glycocylic nature of rutin, this compound tends to be located in the interfacial region of lipid bilayers in liposomal systems. Additionally, the P=O (phosphate) and C=O (carbonyl) groups of lipids are believed to aid in the formation of hydrogen bonds with the OH groups of sugar and the main ring [120,121,122,123].

In research studies using the fluorescence emission spectrum, rutin was incorporated deeply into the lipid bilayer of liposomes formulated from phospholipids extracted from egg yolk. As a result, the authors concluded that despite its polar 3-O-rutinose substitution, rutin is capable of forming bonds with the hydrophobic chains of phospholipids. In addition, it was found that liposomal formulations reduced the formation of reactive oxygen species; therefore, these systems become good candidates for in vitro and in vivo neuroprotective applications [109].

On the encapsulation of glycosylated flavonoids such as rutin in liposomes, fluorescence and FTIR spectroscopy were used to determine that the OH groups of the sugar were attached to the P=O groups of the lipids, while the OH groups of the ring were in close proximity to the C=O lipid groups [119].

Liposomal systems have been developed as vehicles for specific organ-targeted delivery of nutraceuticals or cosmetics. To improve the selectivity, liposomes are coated with natural or synthetic polymers, depending on the intended use. Common natural polymers, such as arabic gum, pectins, alginate, chitosan and maltodextrin, are often used to encapsulate rutin. Table 2 shows research related to rutin encapsulation in liposomal systems.

#### 6.1.2. Nanoemulsions

Nanoemulsions have been used to preserve the biological activity of plant extracts that are rich in rutin. Natural antioxidants are susceptible to chemical degradation caused by factors such as temperature, oxygen and pH. Additionally, the limited stability, low solubility and imparting of taste, odor and color of plant extracts when applied directly in a product pose a challenge. Therefore, nanoemulsions are formulated to address these challenges [115,124].

Nanoemulsions are stable, heterogeneous colloidal systems consisting of small, spherical droplets of a dispersed phase within a liquid phase. The two liquids are typically immiscible, with an oily phase and an aqueous phase, both covered by a layer of surfactant components that provide physicochemical stability. The droplet size of nanoemulsions is typically around 100 nm [112,125,126,127].

In the formulation of nanoemulsions, the energy to expand the interfacial surface is higher and positive compared with the small entropy of emulsification, which can lead to phase separation during long storage periods. As a result, the phenomena of creaming/sedimentation and flocculation are ignored, and only the mechanisms of coalescence and Ostwald ripening are considered in relation to the instability of nanoemulsions [112,125].

Nanoemulsions can be formulated using emulsification methods with either high or low energy, and are classified based on the specific physicochemical phenomena involved. Characteristics such as polarity, solubility, viscosity and the density and concentration of the oily phase and complementary components all contribute to the stability of nanoemulsions; this determines important factors such as droplet size, polydispersity index and Z potential.

The efficacy of rutin formulated in nanoemulsions was studied to verify its ability to scavenge free radicals by binding to antioxidant enzymes in the brain and causing their activation, thereby reversing free radical-induced oxidative stress, which is associated with Parkinson’s disease [128].

Nanoemulsion containing *Physalis peruviana* calyx extract which includes the compound rutin was developed. The extract has a low solubility, which limits its industrial applications, so encapsulation techniques have been studied to improve solubility. The nanoemulsion was found to contain 11 μg/mL of rutin and exhibited a droplet size of 160–180 nm with an encapsulation efficiency of 85%. Stability was evaluated for 120 min through the reducing capacity [129]. However, the study reported a loss of 28% of rutin, which is comparable to other studies of plant extracts [115,130]. Table 2 provides additional information on the formulation methodologies for rutin-loaded nanoemulsions as well as their main results.

#### 6.1.3. Lipid and Biopolymeric Nanoparticles

Lipid-based nanoparticles and biopolymeric nanoparticles are a set of nanocarrier systems formulated for the incorporation of bioactive compounds, mainly hydrophobic compounds [131].

Lipid-based nanoparticles consist of two subgroups referred to as solid lipid nanoparticles and nanostructured lipid carriers [132]. The former comprises lipophilic nanostructures formed from biocompatible, completely solid lipids such as triglycerides, glyceride mixtures or waxes at room temperature [133]. Solid lipid nanoparticles have several benefits, including encapsulation advantages like stability, sustained release of bioactive compounds and biocompatibility. However, they also have some drawbacks, including low loading capacity, unexpected gelling behavior and the possibility of expelling the encapsulated substance or compound during storage due to lipid rearrangement [132].

Nanostructured lipid carriers, unlike solid lipid nanoparticles, are formulated using a combination of solid and liquid lipids, with the solid lipids comprising a higher percentage of the mixture. These systems have a foundation of amorphous crystals within their structure, which allows for greater space to incorporate bioactive molecules and reduces the risk of expulsion during storage [134].

Both solid lipid nanoparticles and nanostructured lipid carriers offer advantages in formulation, but ongoing research aims to address the challenges and identify the ideal combination of materials to improve stability under adverse conditions. As potential candidates for use in food or pharmaceutical applications, nanostructured lipid carriers offer advantages such as improved bioavailability and dispensability in aqueous formulations. However, the presence of hydrophilic compounds in lipid nanoparticles can lead to system instability due to the partitioning behavior of the payload molecules in the aqueous medium during preparation.

Biopolymeric nanoparticles are solid structures with sizes ranging from 1 nm to 1 μm. These structures can be classified as either nanospheres or nanocapsules, depending on their structure. Nanospheres are matrix-type systems that can encapsulate bioactive compounds within the center of the matrix or adsorb them on the surface, while nanocapsules are vesicular systems with an oil core surrounded by a polymeric membrane. These types of structures have the advantage that the molecule or compound to be encapsulated can be dissolved in the center of the core or adsorbed on the surface of the capsule, depending on the purpose of use [131].

Ionic gelation and nanoprecipitation are common methods for producing lipid and biopolymeric nanoparticles. On the other hand, hydrophobic polymers are formulated using organic solvents in a process that involves solvent drag or displacement methodologies, followed by polymer nanoprecipitation assisted by water-soluble solvents.

Rutin was formulated using bovine milk proteins and trehalose through coprecipitation and lyophilization. An acidic pH was induced to facilitate precipitation, with the objective of improving the solubility of rutin through a simple and biocompatible formulation. The results indicated a significant improvement in the solubility and dispensability of the precipitate under alkaline conditions, which could be partially maintained after subsequent precipitation under acid conditions. These findings suggest the solubility of flavonoids could potentially be improved under alkaline conditions, as some of the hydroxyl groups of rutin can become acidic at a pH above neutral [135].

The plant extract from cape gooseberry (*Physalis peruviana*) calyx was evaluated to formulate microparticles using hypromellose phthalate as the polymer. The formulation contained rutin as an analysis marker. Results showed that the entrapment efficiency was 71% and yield was 64%. A glucose tolerance test further demonstrated substantial hypoglycemic activity of the encapsulation method. Furthermore, considering that rutin is highly labile to acid hydrolysis, the encapsulation method was shown to release only 8% of rutin at an acidic pH. These findings suggest the encapsulation method effectively protects rutin from degradation through acid hydrolysis [110]. Table 2 complements the information about rutin encapsulation by lipid and biopolymeric nanoparticles.

**Table 2:** Rutin encapsulation in colloidal and heterodisperse systems.

Encapsulation System	Wall Materials	Encapsulation Mechanism	Package Characteristics	In Vitro Release	Ref.
Anise extract nanoemulsion (extract with rutin content)	The extract (5 mL) was added into 10 mL H_2_O containing between 5% and 80 (with respect to extract weight)	Ultrasound 3 min, 50% amplitude, under ice cooling, agitation 24 h at room temperature	Droplet size: 400 nm, polydispersity index: 0.23, after 6 months the average droplet size increased to 649 nm	--	[136]
Nanoemulsion	7% of soybean oil (*w*/*w*), yarrow extract solution (1 mg/mL), 2% sodium caseinate as emulsifier	Homogenization 30,000 rpm 2 min, high pressure homogenization 450 kPa, five passes	Droplet size: 248 nm, Z potential: −37.9 ± 0.7, stability: two weeks	Nanoemulsion partially protected yarrow phenolic compounds during digestion; after digestion, phenolics in milk gels showed the highest antioxidant activity	[137]
Rutin emulsions stabilized by chitosan and lecithin	Continuous phase: lecithin (5% *p*/*p*), chitosan and water, the dispersed: rutin was dissolved in soybean oil (0.1% *w*/*w*). Chitosan solutions 2% (*w*/*w*)	Spontaneous emulsification followed by rotor-stator homogenization	Emulsions flow curves showed a near-Newtonian behavior, droplet size: 520 nm, stability: 30 days	Thermal degradation followed first-order reaction kinetics. The activation energy value for rutin degradation was 27.8 kJ mol^−1^.	[138]
Lipid carrier	Lipid phase: 100 mL olive oil, 15 mL oleic acid, 11.5 g rutin; the aqueous phase: 6.9 g of lecithin in 115 mL distilled water.	Homogenization at 1700 rpm 20 min followed by ultra-sonication 40 kHz, 30 min	Encapsulation efficiency 99.85%, particle size: 1.7 μm, lipid carrier significantly increased the ABTS radical scavenging ability, singlet oxygen-scavenging ability and lipoxygenase inhibition	Highest radical inhibition activity for all the digestive phases, controlled release (60 h)	[139]
System (SEDDS) for rutin fatty ester	The rutin fatty ester: (SEDDS to 7% *w*/*w*); polymer: [dimethylsiloxane-co-(3-(2-(2-hydroxyethoxy) ethoxy)propyl]methylsiloxane] (10%), Transcutol HP 40% *w*/*w*, Cremophor RH, 40% *w*, Labrafac PG 20% *w*/*w*	Self-emulsifying delivery system (SEDDS); enzymatic acylation rutin: lauric acid, catalyzed by lipase from Candida antarctica in acetone.	Droplet size: 48.4 nm, the incorporation of 10% of the polymer in SEDDS showed an almost 2-fold increase in mucus permeation	log D value of 3 indicating sustained release of the rutin ester	[140]
Rutin-NaCas co-precipitates	Sodium caseinate (10% *w*/*v*), rutin (10% *w*/*v*), trehalose (0, 2.5 or 5% *w*/*v*), the solution was acidified using a 4 M HCl	Lyophilization followed by co-precipitation	Entrapment efficiency: 98.1%, loading capacity: 48.6%, the addition trehalose improved the dispersibility and solubility of precipitate	--	[135]
Polycaprolactone-based nanocarrier	Organic phase: Rutin, Polycaprolactone, acetone (solvent), aqueous phase: Poloxamer 407, Polysorbate 80	Nanoprecipitation technique	Particle size: 173.63 nm, polydispersity index: 0.107, zeta potential: −22.63 mV, encapsulation efficiency: 72.64 ± 1.06%	Sustained in vitro release for 48 h (65.54–73.74%)	[141]
Rutin nanocomplexes	Rutin: Dimethyl sulphoxide (2.5% *w*/*v*), phosphatidylcholine: t-butyl alcohol (1.5% *w*/*v*). Rutin: phosphatidylcholine ratios (1:2 and 1% *w*/*v*) mannitol as a cryoprotectant	Solvent evaporation, salting out and lyophilization	The in vivo study showed better hepatoprotective activity of the formulation compared with pure rutin with improved oral bioavailability	Rutin nanocomplexes significantly improved the solubility and in vitro drug release, and kinetic studies confirmed the diffusion-controlled release	[22]
Rutin-loaded starch nanospheres	10 mL of the rutin standard, 20 mg of the starch, Dimethyl sulphoxide (10 mL)	Dialysis embedding method, bag (MWO = 8000 to 15,000 g/mol)	The drug loading rate: 0.43 μg/mg, encapsulation efficiency: 85.7%, particle size: 70.16 nm	Controlled release, the rutin release rate was approximately 75.38% in pH 7.2	[142]
Rutin Nanocarrier	1. Rutin: ferritin (molar ratio of 28.2: 1). 2. Epigallocatechin gallate+Rutin/ferritin (binding number: 27.30, binding constant K: 2.65 × 10^−4^ M^−1^)	Homogenization, microfiltration, dialysis (MW 10 kDa cutoff)	Encapsulation efficiency: 18.80%, loading capacity: 2.98%, improved rutin stability	Prolonged release in simulated gastrointestinal tract fluid: Release rate of 47.1%	[143]
Liposomes	Phosphatidylcholine (10 mg/mL) and rutin (5 mg/mL) Glycerol (3%)	Heating/homogenization method	Particle size: around 419 nm, zeta potential: −40 mV, suspension stability: more than 30 days	Continuous process during the testing period (72 h).	[144]
Rutin-phospholipid nanoliposomes (Egg yolk phospholipid extracts)	(Lipid 25 μM + rutin 16.7 μM). Phospholipid content in the final fraction: 208.65 μmol/g fresh egg yolk, cholesterol (0.069–0.082 cholesterol/phospholipid molar ratio), lutein and zeaxanthin (89.24 and 14.9 mg/g, respectively). Saturated fatty acids: 50% of egg’s total yolk phospholipids, monounsaturated fatty acids: 20 to 25%, and polyunsaturated up to 35%.	Thin-film hydration method, followed by sonication cycles (2 min) alternated with hand shaking (2 min), for a total of 20 min at (37 °C)	Mean diameter < 140 nm, entrapment efficiency of rutin up to 87%, rutin-liposome attenuated glutamate-induced cytotoxicity	--	[109]
Cationic liposomes	DOPC (1,2-dioleoyl-sn-glycero-3-phosphocholine), DOPE (1,2-di-(9Z-octadecenoyl)-sn-glycero-3-phosphoethanolamine), final ratio lipids and antioxidant 1:1	Thin-film hydration method	Particle size: 129 nm, polydispersity index: 0.16, zeta potential: 35, stability: 3 months storage	Rutin liposomes interfere with the hydrogen peroxide-induced toxicity, showing a good ability as cell protector.	[145]
Rutin phytosomes	Rutin in methanol (40 mg/mL), 1,2-Dipalmitoyl-sn-glycero-3-phosphocholine (10 mg/mL), ratio of methanol in tetrahydrofuran (less than 10% *v*/*v*)	Thin-film hydration method followed by: bath sonication 40 min, extrusion through 200 nm and then 100 nm pore-size membrane filters	Mean diameter less than 200 nm neutral charge, the majority of the rutin is likely to be associated with lipid headgroups either in the core of the phytosome or on the outer surface of the phytosome (FT-IR and DSC analysis)	The release was less than 20% in all biorelevant media, release depends on membrane diffusion. Maximum release (over 2 h 10.4% and 15.9%)	[146]
Liposomes (carboxymethyl cellulose edible films)	Dipalmitoyl phosphatidylcholine (1 mg/mL), cholesterol 0.20 mg/mL), rutin (0.3 mg/mL), solvent: chloroform/methanol (3:1 *v*/*v*)	Thin-film hydration method followed by extrusion 10 times through polycarbonate filters 400 nm nominal pore size. Carboxymethyl cellulose edible films were prepared with casting method	Polydispersity index: 0.20, encapsulation efficiency: 74.1%, zeta potential 36.9–42.4 mV, stability: 21 days 4 °C	Controlled release (21 h) flavonol-loaded liposomes were incorporated into carboxymethyl cellulose edible films	[147]
Rutin nanophytosomes	Molar ratio rutin: phosphatidylcholine (1:3)	Thin-layer hydration method	Particle size < 100 nm and encapsulation efficiency: 99%, physical and chemical stability (30 days of storage)	--	[148]

## 7. Rutin, Industrial Applications

### 7.1. Rutin in the Food Industry

In the context of the food industry, the study of phenolic compounds is focused on the standardization of transformation processes that avoid the degradation of the compound, the substitution of chemical additives or the potentiation of the nutritional contribution of foods and the design of bioactive packaging. Although the multiple health benefits of rutin are widely known, its application in the food industry is still scarce and restricted due to the physicochemical characteristics of rutin in aqueous media and the bitter astringent taste characteristic of phenolic compounds. In addition, this is also because during processing such as cooking, rutin is susceptible to degradation.

Among the usual preparations, cooking is a method that has been monitored to assess rutin content. The rutin content in asparagus cooked between 80 and 90 °C was evaluated; it was found that at temperatures below 90 °C and times of 13 min, there was no significant degradation of the rutin compound [149].

The conditions for retaining the maximum amount of rutin in the dough obtained from buckwheat were studied. Their aim was to inactivate the enzymes that degrade rutin during heat treatment, which generates a bitter taste. Hydrothermal treatment at 50 and 70 °C was found to be optimal for enzyme denaturation in any buckwheat material. The dough produced with the optimal parameters was considered a rutin-rich functional food ingredient [150].

In the same line of research, paste from buckwheat was prepared, and it was found that samples containing hydrothermally treated paste (70 °C, 50 min) showed a higher rutin content (0.83 g/100 g) than the control paste (0.27 g/100 g). Additionally, the hydrothermally treated paste presented lower sticking parameters and lower viscoelastic properties [151].

Rutin and plant extracts containing it have been utilized in meat-product formulations to evaluate their antioxidant efficacy. A commercial antioxidant used in the formulation of sausages was replaced by a mixture of phenolic compounds, 30% of which were rutin. Results demonstrated oxidative stability at 45 days, as measured by the concentration of malondialdehyde [152]. In another study, a meat formulation enriched with buckwheat husk extract with a high concentration of rutin demonstrated stability after 180 days of storage, greater ability to control free radical scavenging activity measured by 2,2-diphenyl-1-picrylhydrazyl and greater Fe(II) ion chelation capacity [153].

### 7.2. Active Packaging

Rutin formulations have been studied for making containers and packaging. Dipalmitoyl lecithin liposomes were formulated to encapsulate quercetin and rutin, which were then incorporated into edible carboxymethylcellulose films. The physicochemical characterization reported a low polydispersity index (0.32 and 0.20), high encapsulation efficiency (88.9 and 74.1%) and a Z potential of 42.4 mV. It was observed that liposomes were appropriate for encapsulating rutin and controlling its release. In addition, the antioxidant efficacy of the flavonol rutin was demonstrated [147].

On the other hand, edible bioactive films were developed using corn starch by incorporating zein and rutin nanoparticles. Nanoparticles at a concentration of 10% *w*/*v* produced the best characteristics of antioxidant activity. However, the addition of nanoparticles at concentrations greater than 10% increased the tensile strength and elongation of the starch films. Additionally, the incorporation of zein and rutin nanoparticles into the films was found to decrease the water vapor permeability and water solubility compared with films without incorporated nanoparticles. The study also found the rutin present in the films was released in controlled conditions and the concentration of the compound between 27.1% and 36.9% was achieved after 12 h [154].

### 7.3. Food Fortification

The enrichment of food matrices with bioactive compounds presents a significant research challenge, and while there is still a shortage of studies on the topic, there has been an increase in publications related to it. Rutin and quercetin at a concentration of 0.5 g/100 g were added to a spreadable cheese formulation and the effects of temperature and melting time (80 and 90 °C for 1.5 and 10 min) were evaluated on the retention of quercetin and rutin, total phenolic content and antioxidant capacity. The study found the content of the two flavonoids significantly decreased, affecting antioxidant capacity. Melting temperature had a greater impact on rutin than quercetin [155].

Food-grade nanostructured lipid carriers were developed to encapsulate rutin and incorporate them into milk, orange juice and apple juice. The oily phase was composed of oleic acid and cocoa butter, and surfactants were added. A 10% rutin/lipid ratio was found to be optimal. The food models were exposed to pasteurization (65 °C for 30 min) and sterilization (121 °C for 15 min) conditions, and the results showed the fortification did not have a significant effect on the final product. The stability of the particle size, pH and turbidity were evaluated over 30 days, and the properties of the enriched foods were not adversely affected [156].

### 7.4. Cosmetic Applications

Natural substances with photoprotective potential and antioxidant activity have gained interest in recent years due to their ability to provide added benefits in conventional sunscreen formulations. The clinical safety, antioxidant capacity and sun protection factor (SPF) of rutin in sunscreen formulations with 0.1% and 3% *w*/*w* rutin were evaluated. Their findings revealed that the formulation with 3% rutin was capable of increasing the SPF up to 70%, indicating that it was effective and safe to use in sunscreen formulations. This formulation was also found to increase the elimination of free radicals by 40% compared with formulations without rutin [157].

The effects of rutin on the disruption or lysis of the membranes of cutaneous fibroblasts induced by UV rays were investigated. The fibroblasts were incubated for 12 h and then exposed to UVA and UVB radiation for 24 h. Lipidomic analysis showed that rutin reduced the level of phosphatidylethanolamine and phosphatidylcholine and prevented the increase in dospholipase, as well as prevented the formation of reactive oxygen species. Furthermore, it prevented the decrease in levels of glutathione peroxidase and vitamins E and C, suggesting its potential as a photoprotective and antioxidant agent for skin care [158].

The potential benefits of rutin-containing dermocosmetic products were evaluated in terms of their absorption in the UVA range and their ability to eliminate reactive oxygen species. Tests were conducted on different formulations, using methods such as antioxidant activity, in vitro photoprotective effectiveness, photostability and in vivo skin tolerance. The results suggested good compatibility with human skin and an antioxidant potential with elimination activity values 75% higher than typical UVB filters. Although rutin could not prevent post-irradiation photodegradation, it did show a significant increase in the length of wavelengths, especially in the UVA range, which indicates a photoprotective gain [159].

As part of the broader study, the antioxidant capacity and potential sun protection benefits of rutin-loaded gelatin nanoparticles were studied. It was found that the antioxidant capacity increased by 74% for encapsulated rutin in these particles in comparison to free rutin solution. Additionally, the sun protection factor (SPF) improved by 48%. The researchers also performed skin compatibility tests, which demonstrated good performance. These findings suggest that rutin-loaded gelatin nanoparticles have the potential to serve as an effective and biocompatible photoprotective agent with added antioxidant benefits [160].

## 8. Discussion

Fruit and vegetable crop production using family farming approaches has become more common as a means of addressing per capita consumption. These crops have gained significance in daily diets due to their nutrient content and biological value, and also provide employment opportunities for local communities. Fruit and vegetable foods are currently being utilized worldwide to address malnutrition, poverty and issues related to economic prosperity. Increasing the consumption of fruits and vegetables is vital to enhance food security and nutrition [161]. Moreover, incorporating non-animal food products, including fruits and vegetables, is crucial to building sustainable and innovative food systems [162]. Over the last six decades, fruit and vegetable production has increased by over 30% [161], highlighting the essential role of these crops in global food systems.

The study of antioxidants from natural sources has become a rapidly expanding field in response to the global trend towards safer and more sustainable food additives. While numerous investigations have reported on the biological activities of antioxidants as pure chemical compounds, much less is known about the antioxidant content of everyday foods such as fruits and vegetables. In order to fully understand the potential health benefits of plant species, it is crucial to have accurate and comprehensive knowledge of their molecular composition. Despite multiple investigations, the technology for extracting, identifying and quantifying antioxidant compounds from plant sources is not yet widely available.

The updated bibliographic search for this review has shown that, although the Soxhlet methodology remains the most frequently used technique for rutin extraction, increasing attention is being paid to new and promising extraction techniques that promote efficient recovery of this valuable phenolic compound while reducing the use of solvents, time and energy. Accordingly, research efforts hope that the results do not compromise the composition and biological properties of rutin and that the achieved yield is close to that of conventional techniques. This trend reflects a growing emphasis on sustainable and eco-friendly solutions for the extraction of bioactive compounds, driven by the desire to minimize environmental impact and support the development of green technologies.

Antioxidant compounds such as rutin have been incorporated into encapsulating matrices under nanotechnology approaches with the aim of providing biological characteristics to food, cosmetic and nutraceutical formulations. However, despite the significant advances in rutin encapsulation, there remain several challenges that limit the industrial scalability of these approaches. As highlighted in this review, there are still significant obstacles to overcome in terms of high production costs and limited industrial applications for rutin encapsulation systems without neglecting parameters that are a challenge for research, such as the release of the compound in a specific area, the absorption in the intestine after release, the interaction with food ingredients and the interaction with the intestinal microbiota [163].

It is important to emphasize the significance of researching new raw materials that have compatible properties with rutin and the matrix in which it will be applied. Additionally, it is crucial to use materials that reduce adverse effects and the accumulation of waste in the environment. Despite the use of techniques that involve low energy expenditure to achieve small sizes in the range of micrometers or nanometers, the use of chemical solvents is still needed to increase the physicochemical stability of the encapsulated system. As a result, a synergy between formulation techniques is necessary to advance research into this compound.

Generally, the technology for the formulation of rutin encapsulation systems is well-established; however, its practical applications are limited. In future research, it is necessary to explore nutraceutical or food grade formulations, as this approach remains understudied for the rutin compound but is essential in the industry, where synthetic additives have less acceptance by consumers, particularly in food preservation, food quality and functional foods.

## 9. Conclusions

In recent years, there have been reports of rutin extraction from natural sources, including native plants from different regions worldwide. In this review, studies have been compiled presenting the extraction mechanisms and the rutin content present in fruits and vegetables commonly included in a normal diet, as well as those cultivated through family farming approaches. The information presented can be used as a reference for future research related to rutin extraction and encapsulation, with a view to promoting food safety and assessing phytonutrients such as rutin. Despite not being classified among the macronutrients, rutin is a compound with valuable biological activity and which provides health benefits.

Rutin has been used as a study compound to evaluate encapsulation mechanisms due to its ideal structural configuration for studying hydrophilic or lipophilic wall components. This feature permits the investigation of the synergy between the formulation components and, thus, facilitates the exploration of new industrial formulations.

Despite the description of methods of rutin encapsulation, the potential applications of this compound in various sectors, such as the food, cosmetics and pharmaceutical industries, remain underexplored; however, it offers alternatives for the industrialization of both the pure compound and plant extracts. Given the continuous growth of these industries, further research efforts are necessary in this area. It is also vital to study the incorporation of encapsulated rutin in practical formulations, articulating the current findings with new investigations with pilot- and industrial-scale approaches.

## Data Availability

Not applicable.
